# The Origin of the “Seasons” in Space Weather

**DOI:** 10.1038/s41598-017-14957-x

**Published:** 2017-11-07

**Authors:** Mausumi Dikpati, Paul S. Cally, Scott W. McIntosh, Eyal Heifetz

**Affiliations:** 1 High Altitude Observatory, National Center for Atmospheric Research, 3080 Center Green, Boulder, CO 80301 USA; 2 School of Mathematical Sciences, 9 Rainforest Walk, Monash University Clayton, Victoria, 3800 Australia; 3 Department of Geoscience, Tel Aviv Univ., Tel Aviv, 69978 Israel

## Abstract

Powerful ‘space weather’ events caused by solar activity pose serious risks to human health, safety, economic activity and national security. Spikes in deaths due to heart attacks, strokes and other diseases occurred during prolonged power outages. Currently it is hard to prepare for and mitigate the impact of space weather because it is impossible to forecast the solar eruptions that can cause these terrestrial events until they are seen on the Sun. However, as recently reported in Nature, eruptive events like coronal mass ejections and solar flares, are organized into quasi-periodic “seasons”, which include enhanced bursts of eruptions for several months, followed by quiet periods. We explored the dynamics of sunspot-producing magnetic fields and discovered for the first time that bursty and quiet seasons, manifested in surface magnetic structures, can be caused by quasi-periodic energy-exchange among magnetic fields, Rossby waves and differential rotation of the solar interior shear-layer (called tachocline). Our results for the first time provide a quantitative physical mechanism for forecasting the strength and duration of bursty seasons several months in advance, which can greatly enhance our ability to warn humans about dangerous solar bursts and prevent damage to satellites and power stations from space weather events.

## Introduction

Recent observations indicate that variability within a sunspot cycle consists of quasi-periodic bursts of activity followed by quieter intervals; these are called the “seasons” of solar activity^1^. Complete intervals of burstiness followed by quiet are 6–18 months in duration; bursts often persist at certain longitudes for several rotations. These bursts include enhanced coronal mass ejections (CMEs) and flares from which magnetic fields and energetic particles travel through interplanetary space to interact with the Earth, producing ‘space weather’. Currently space weather events, hazardous to our technological society, are predicted only after the CMEs and flares have been observed at the Sun, leaving only a few days to prevent disruptions of power grids, radio communications and global positioning systems (GPS). Numerous industries rely on GPS for accurate orientation and navigation. On flights over polar regions during space weather events, airplanes can experience radio blackouts and equipment disruptions, and satellites can go out of control. About three decades ago, a CME about the size of 36 Earths erupted from the Sun’s surface and ripped through the space at a million miles per hour speed. Two days later this huge plasma ball crashed against the Earth’s magnetosphere, and most significantly Canada’s Hydro-Quebec power utility grid crashed, knocking out electricity to six million people for nine hours.

Extreme space weather events can have devastating effects on human safety and health, as well as cause major damage to economic activity^2^. Extended power outages due to space weather can disrupt water and waste water distribution systems, cause loss of perishable food and medications, shut down heating and air conditioning, computer and telephone systems, public transportation, fuel distribution and pipelines and any system that does not have back-up power. Increased deaths from stress-induced heart attacks, strokes, asthma and other diseases have occurred. National security surveillance systems can be compromised and false signals of impending attack created. Much of the damage and loss of life could be prevented if there existed ability to forecast extreme space weather events much further into the future than possible now. No method exists to make a reliable prediction of when, or where, the next burst of solar activity will occur. Global organization of these persistent, longitude-dependent, oscillating magnetic signals suggests their origin lies in the solar tachocline^3^, a thin shear-layer that separates the solidly rotating radiative core and differentially rotating convective envelope.

**Figure 1:**
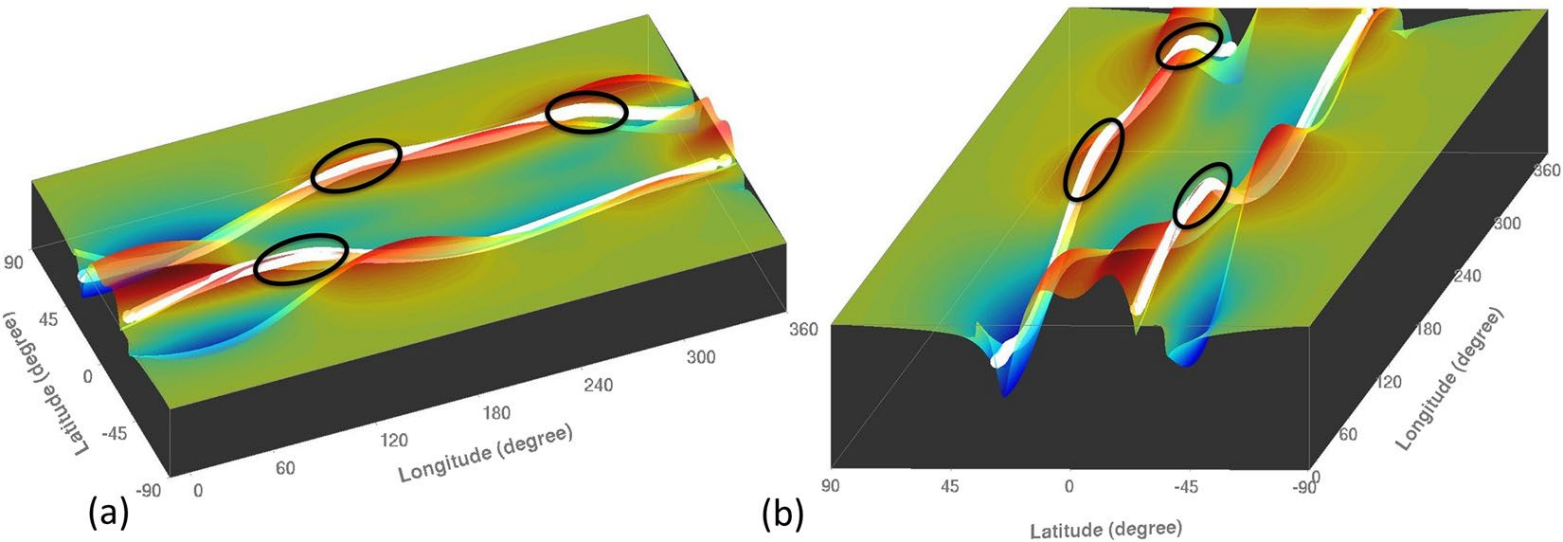
Two perspective snapshots of top-surface (color-shade) of a tachocline fluid shell, viewed respectively along longitude (left panel) and latitude (right panel), during its MHD evolution; red/orange represents swelling of the fluid and blue/sky-blue the depression. Yellowish-green represents neutral thickness. The shallow-water tachocline model has a rigid bottom and deformable top; vertical extent denotes the tachocline thickness (20 times enlarged). Portions of the toroidal magnetic bands (two white tubes one each in the North and South hemispheres) that coincide with swelled fluid are shown encircled by black ellipses – these portions start entering the convection zone, and hence are more likely to buoyantly erupt at the surface.

The solar tachocline straddles the base of the convection zone. Turbulence from convection in this zone declines rapidly with depth. Its global dynamics is plausibly 2D or quasi-3D. Extensive studies of global hydrodynamics (HD) and MHD of the solar tachocline in 2D and quasi-3D shallow-water type numerical models show persistent patterns of bulges and depressions of tachocline fluid with low longitudinal wave numbers^4–9^. Portions of the solar dynamo-generated, sunspot-producing, toroidal magnetic bands that coincide with bulging of the fluid are likely to emerge buoyantly to the surface through the convection zone (see Fig. 1). This bulging leads to magnetic flux emergence and the enhanced formation of active regions^10^. This commonality of observation and global-scale organization of model-inferred magnetic activity supports the concept that the seasons of space weather originate in the solar tachocline.

The observed quasi-periodic bursts are also known as Rieger-type periodicities of 150–160 days^11,12^, as well as quasi-biennial oscillations (QBO^13^). Zaqarashvili^14,15^ *et al*. suggested, by employing linearized dynamics of an MHD shallow-water tachocline model, that the interactions of oscillatory neutral modes and growing modes can explain the Rieger-type periodicities and QBO. Motivated by these results, Dikpati^16^ developed a nonlinear shallow-water tachocline model to study the evolution of the differential rotation and Rossby wave disturbances with low longitudinal wave numbers that perturb the system. The shallow-water tachocline model-system, like nonlinear fluid dynamics model-systems, is expected to exhibit periodic amplitude-oscillations between the reference-state and perturbations. We hypothesize that the solar seasons can originate from such nonlinear oscillations between tachocline differential rotation and Rossby waves. What is the physical origin of these Tachocline Nonlinear Oscillations (TNOs), and how robust is their periodicity? Does it agree with the observed range of periodicities of solar seasons? Can these seasons be forecast?

## Results

While the existence of Rossby waves in the Earth’s atmosphere has been known since 1939^17^, these waves have recently been detected on the Sun from patterns of coronal bright points^18^, and also in young Sun-like stars^19^. In a dissipationless system, an oscillation between reference state kinetic energy and perturbation kinetic energy should be expected^20^.

**Figure 2:**
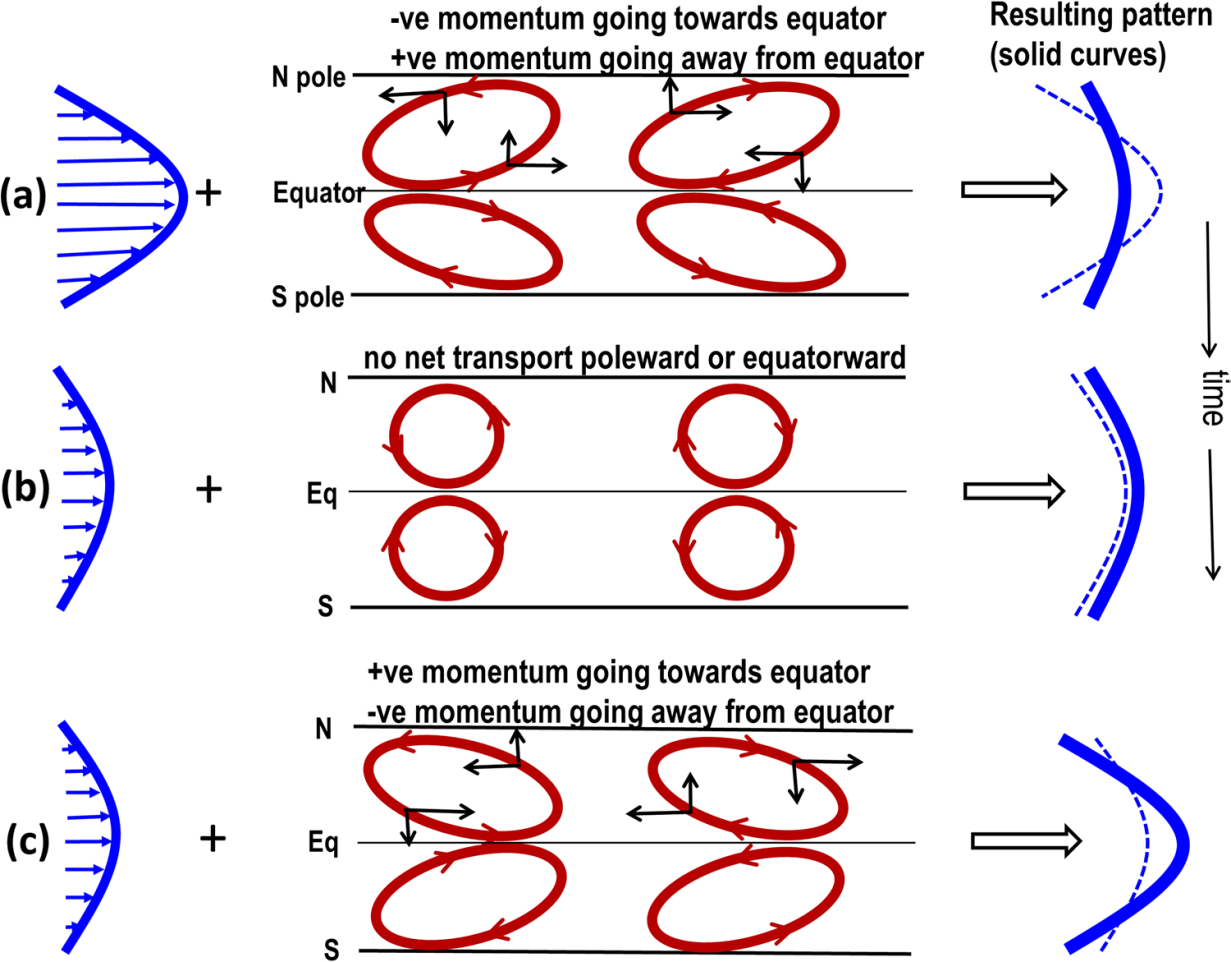
Top, middle and bottom panels (panels a,b,c) show schematically how Rossby waves and tachocline latitudinal differential rotation (DR) interact. Time goes from top panel to bottom. Energy from reference state DR (blue curve in left frame of panel (a)) is extracted by perturbation flow-patterns (eastward-oriented red ellipses in the north hemisphere, plotted in middle frame of panel (a)), because their eastward tilts transport angular momentum away from equator; consequently the polar region spins up and the equatorial region spins down. Thus the pole-to-equator DR decreases (thick blue curve in right frame of panel a) compared to the original (thin dashed curve). Panel (b) shows that tilts become neutral when no more energy can be extracted out of DR. However, the perturbation flow-patterns overshoot from neutral tilt to acquire westward tilts, as shown in panel (c) (middle frame) and hence positive angular momentum is transported back from high to low latitudes (see the black arrows on the flow-patterns of the bottom panel), spinning up the equatorial region again. DR is thus restored back to being the source of energy to be extracted to grow the perturbation flow-patterns. The process repeats, leading to a sequence of tachocline nonlinear oscillations (TNO) between Rossby waves and DR in a nearly dissipationless system.

Figure 2 displays the basic mechanism of interaction between Rossby waves and solar differential rotation (DR). If the mean shear flow in the rotating frame is unstable, Rossby waves grow at the expense of the mean flow by being tilted against the shear, increasing their kinetic energy by decreasing the kinetic energy of the DR, via the Reynolds stress. In the subsequent nonlinear phase, when Rossby waves reach finite amplitudes, the shear is no longer able to provide energy to the waves. The waves weaken, become tilted with the DR and feed back energy to the mean flow, creating an oscillation between Rossby wave and DR; in fluid mechanics a similar oscillation is also referred to as the nonlinear Orr mechanism^21^.

The TNO can give rise to the periodicity in the bursts of activity, through oscillations in the upper boundary of the tachocline–the bulges and depressions. The tachocline toroidal magnetic flux can start its buoyant rise through the convection zone at those times when the tachocline fluid shell has its maximum upward swelling locally^10^, helping the magnetic fields enter the convection zone from the tachocline. Tachocline fluid reaches maximum thickness when the Rossby waves have extracted the maximum possible energy from the differential rotation; thus the quasi-periodic activity bursts can occur to produce the peak of a season of solar activity when the energy of the Rossby waves is maximum. The bursty phase then declines producing the quieter “season” when the differential rotation is restored.

Since the tachocline is so thin (thickness being no more than ~4% of solar radius), employing a shallow-water model for studying the HD/MHD tachocline instabilities is quite appropriate. Hydrodynamic shallow-water models^22^ have been applied in numerous geophysical systems since 1970s. Such models of the tachocline were developed during the past decade since the introduction of MHD shallow-water equations by Gilman^6^, for analysis of global MHD instability solar tachocline latitudinal differential rotation^7,13,15,23,24^, and other astrophysical phenomena^25–27^. Here we investigate the nonlinear evolution of shallow-water tachocline dynamics as a function of the parameters of the model, such as the “effective gravity” (*G*_*eff*_) of the tachocline, the amplitude of differential rotation, the strength of the peak toroidal magnetic field and the latitude location of the peak field. Note that the effective gravity is reduced from the actual gravity (*G*_*actual*_), by the fractional departure of the tachocline stratification from adiabatic.

In linear studies of global, shallow-water tachocline instabilities, a typical latitude-longitude disturbance planform of the eigenfunction of an unstable mode shows eastward tilts^7^ as seen schematically in the first panel of Fig. 2. This tilt implies poleward angular momentum transport by the Reynolds stress, which drives the instability.

**Figure 3:**
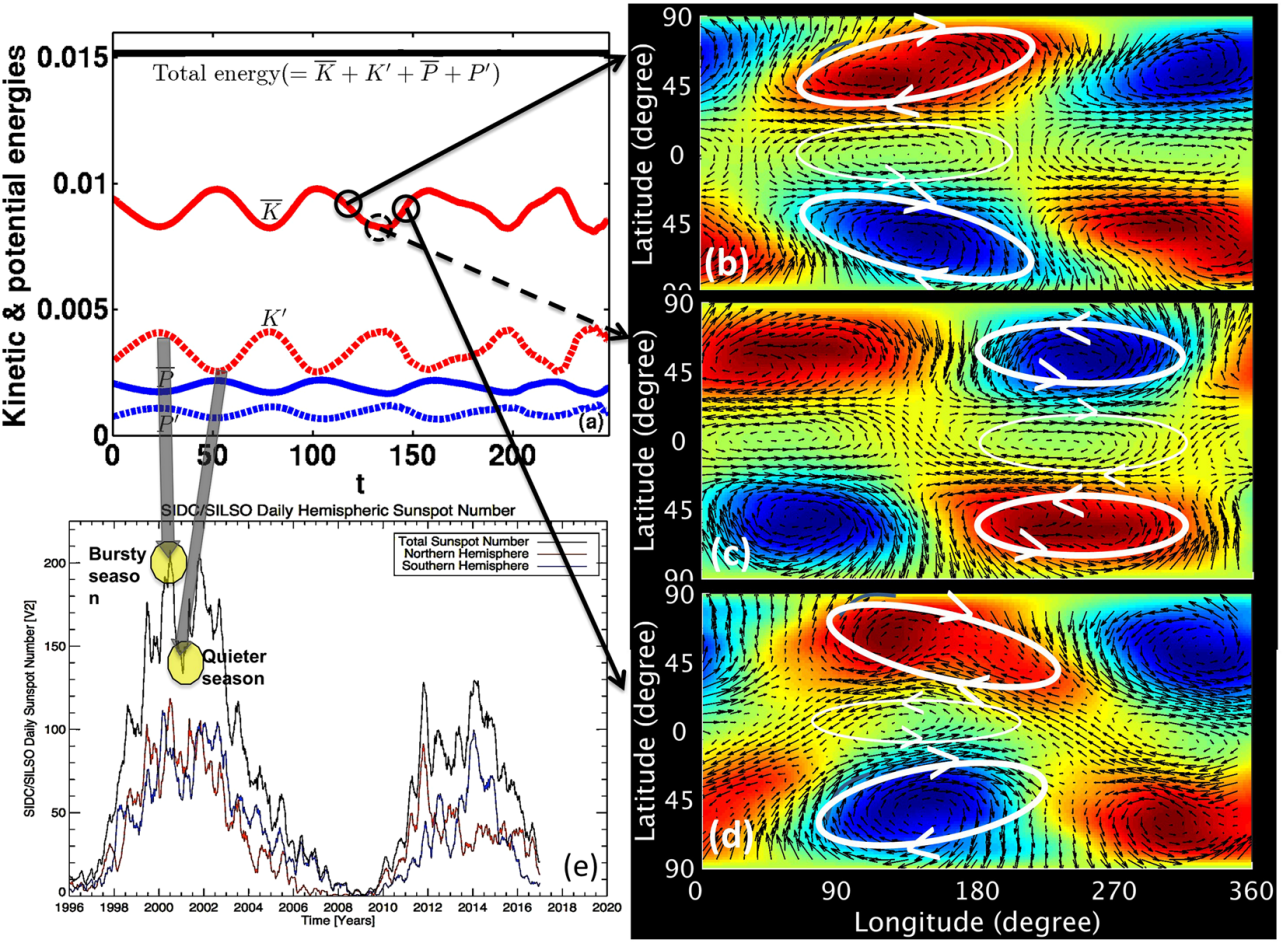
Left panel (**a**) shows oscillation between differential rotation kinetic energy ($\bar{K}$, solid red curve) and perturbation Rossby waves kinetic energy (${K}^{^{\prime} }$, red dashed curve). Typically up to 42 modes in longitude were included in these nonlinear simulations. The units in x and y axis are dimensionless. 100 dimensionless time units correspond to approximately one year; thus the TNO has a period of about 6 months in this case. Frames (**b**–**d**) show perturbation flow patterns (in arrow vectors), and thickness of tachocline fluid shell (in color shades, red shade representing swelling, blue depression). Tilts of perturbations are eastward in (**b**), extracting energy from differential rotation until Rossby waves’ energy is at a maximum; perturbations then go through neutral tilts (**c**), and then overshoot to acquire westward tilts (**d**). Enhanced bursts of activity (shown by a semi-transparent gray arrow pointed towards a local peak (yellow-filled ellipse) in sunspot number curve in panel (**e**)) occurs when perturbation Rossby wave kinetic energy is at its maximum, followed by a relatively quiet season (second semi-transparent gray arrow pointing towards a local dip (the second yellowfilled ellipse) in panel (e)).

However, in nonlinear evolution, a quasi-periodic exchange of energy occurs between the reference state differential rotation and the Rossby wave disturbances (see Fig. 3). Figure 3(a) shows that the reference state kinetic energy ($\bar{K}$, plotted in solid red) from tachocline latitudinal differential rotation and perturbation kinetic energy (*K*′, the red-dashed curve) from Rossby waves undergo an anti-phase oscillation, *K*′ being at its maximum when $\bar{K}$ is minimum and vice versa. The pole-to-equator differential rotation (*ω*), given by $\omega ={s}_{0}-{s}_{2}{\mu }^{2}-{s}_{4}{\mu }^{4}-{\omega }_{c}$, ($\mu$ is the sine latitude), is chosen to be 21% for this case, by setting ${s}_{2}/{s}_{0}=0.15,\,{s}_{4}/{s}_{0}=\mathrm{0.06.}$ The effective gravity is chosen to be ${G}_{eff}$ = 0.5. We set the initial perturbation kinetic energy (*K*′) at 16% (i.e. ~40% initial amplitude to perturbation velocity) with respect to the reference state differential rotation kinetic energy ($\bar{K})$ to start the simulation. *K*′ is calculated by computing the volume integral of perturbation kinetic energy over the entire domain of the shallow-water model. Similarly the reference state kinetic energy (i.e. energy of the latitudinal differential rotation) is computed in the same way (see, e.g. the method section for details). Reference state and perturbation potential energies ($\bar{P}$ and *P*′) are shown in solid and dashed blue curves respectively. Evolution of total energy ($\bar{K}$+*K*′+$\bar{P}$+*P*′) is shown by the black curve, which is almost constant, demonstrating that the total energy is conserved with high accuracy, with a variation of only a fraction of a percent during 2.5 years simulation.

In Fig. 3(b) we show how the disturbance patterns evolve with time. In the course of their evolution they extract energy from differential rotation ($\bar{K}$) when $\bar{K}$ is maximum, so the Rossby waves have maximum possible eastward tilts (top panel of Fig. 3(b)). Consequently *K*′ increases and $\bar{K}$ decreases until the epoch is reached when no more energy can be extracted from $\bar{K}$. The tilts are not fixed; they evolve from eastward to westward orientation through a neutral pattern, as seen in the middle panel of Fig. 3(b). As a result, *K*′ continues to increase, eventually reaching its maximum, and the disturbance patterns transport back the angular momentum equatorward, rebuilding the reference state differential rotation.

During this nonlinear evolution of energy exchange between $\bar{K}$ and *K*′, another important variation occurs. The top surface of the tachocline fluid shell deforms the most to penetrate upward through the base of the convection zone when *K*′ is largest; this should help tachocline magnetic fields enter the convection zone to trigger their buoyant eruption to the surface, causing the high season of flux eruption events. Eventually, when no more energy extraction from $\bar{K}$ is possible, the high season ceases and a low season appears. TNO period is therefore expected to determine the periodicity of enhanced bursts of activity, and hence the seasons of the solar activity.

The TNO in Fig. 3 has a simple period of ~6 months, because this case is dominated by the nonlinear evolution of a symmetric mode with longitudinal wavenumber 1, which has a substantially larger growth rate than the other possible unstable modes. If several unstable modes are excited with similar growth rates, we find that interaction among modes makes the planform patterns more complex.

**Figure 4:**
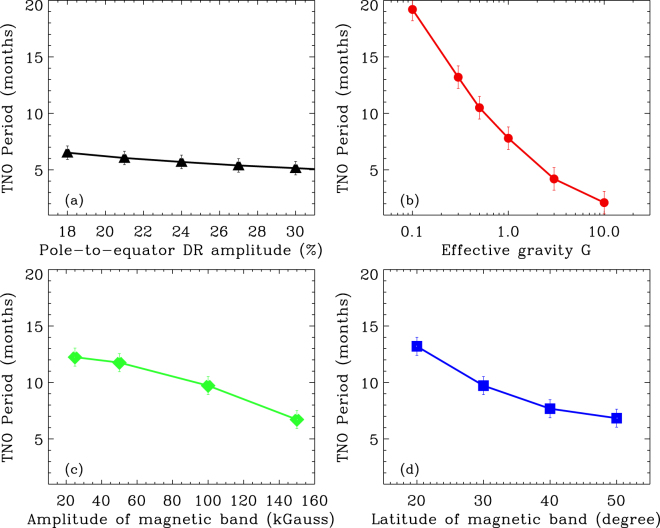
Panels (a,b,c,d) respectively show TNO periods as function of differential rotation amplitude (s), effective gravity (${{G}}_{eff}$), magnetic field strength and latitude-location of magnetic band. Panels (a,b) are produced from a hydrodynamic shallow-water model, in which ${{G}}_{eff}$ = 0.5 in (**a**) and s=21% in (**b**). Panels (c,d) are produced using the MHD shallow-water model with ${{G}}_{eff}$ = 100 and s = 21%; high ${{G}}_{eff}$ is used here to keep the primary interaction between magnetic and kinetic energies, by keeping the potential energy (which is inversely proportional to ${{G}}_{eff}$) low. In all cases TNO periods fall within the range of 2–20 months, indicating the robustness of our results. Error bars are calculated from the variance in periods within each full simulation.

To investigate how robust the TNO periodicity is, we study the nonlinear evolution of shallow-water tachocline dynamics as functions of the parameters of the model, namely the differential rotation amplitude, the effective gravity (*G*_*eff*_), toroidal magnetic band’s latitude-location and its band-strength, with initial perturbations to the system of no more than 16% with respect to the DR energy. Cases with higher and lower than 16% perturbation energy have also been studied, but not shown here. The results are presented in Fig. 4.

The solid curve connecting the triangles in panel (a) reveals that the TNO periodicity is not very sensitive to variation in differential rotation amplitude, whereas the period decreases with the increase in the effective gravity, *G*_*eff*_, of the tachocline fluid-layer, but still in the observed range (panel b). The larger is *G*_*eff*_, the heavier is the fluid, and so the available energies are used up faster, causing smaller deformation of the top surface. Therefore, seasonal bursts of solar activity would occur in faster intervals if high *G*_*eff*_ of the radiative tachocline is dominating the dynamics more than the overshoot tachocline (characterized by low *G*_*eff*_).

TNOs produced from the hydrodynamic model can produce quasi-periodic bursts of activity when the spot-producing toroidal magnetic fields are passively frozen in the tachocline plasma, and therefore not directly participating in the dynamics. If instead, the spot-producing fields actively participate in TNO dynamics, they can affect the resulting TNO periods, which we study by using an MHD shallow-water model^23^.

Panels (c,d) present two examples of modified TNO periods due to the spot-producing toroidal magnetic bands that actively participate. We examine this by including a Gaussian toroidal band of 10-degree width in latitude^5,8^, and extensively explore the TNO periodicity as function of peak field strength of the toroidal band placed at mid-latitude. We find that the TNO period decreases with the increase of magnetic field strength, but remains within the observed range. Placing the magnetic band of 100 kGauss peak field at different latitudes we find that the TNO period decreases as the band is placed at lower and lower latitude, down to about 15-degree – as it would happen with the progress of the solar cycle. This implies that the frequency of bursts increases as the cycle progresses through its peak. This finding is strongly supported by observations^28^. Furthermore recent observational analysis of Rieger-type periodicity in the north and south hemispheres indicates that this periodicity is smaller in the hemisphere that is magnetically dominated^29^, reconciling with our results of decrease in TNO periodicity with increasing field strength presented in Fig. 4c.

The range of periodicity of TNO for a wide, solar-like range of parameters, used in our shallow-water tachocline model, is robust, and falls within the observed range, indicating that the TNO plays a crucial role in causing the seasons of solar activity.

## Discussion

We showed that the dynamics of the solar tachocline shear-layer produces nonlinear oscillations (TNO’s) that periodically exchange energy among differential rotation, magnetic fields and Rossby waves. We found that TNO’s in our model occur with periods of 2–20 months, overlapping with the observed periods of solar seasons. Enhanced activity bursts occur when the Rossby wave energy grows to its maximum by extracting energy from differential rotation, because at that time the top of the tachocline is maximally deformed and hence can help the surface-eruption of magnetic fields as active regions. As time progresses, deformation of the tachocline subsides and the differential rotation gets restored; consequently a bursty season is followed by a relatively quiet season.

This TNO mechanism demonstrates the potential for forecasting solar bursty seasons a few months or even a few years ahead of their occurrence. Building on this nonlinear shallow-water model of the solar tachocline, a forecast model-system can be developed by implementing the methodology of data assimilation and updating the model in every few days, when the new data including the information about global patterns of photospheric magnetic fields from magnetograms become available. So we will be able to predict the activity bursts very much in the way the weather forecast models operate – we can expect the accuracy of the forecast to improve as we get closer in time to our target of several months ahead. Rossby waves^30^ as well as principal components of temporal magnetic field variations^31^ over the past few solar cycles can also be used for long-term predictions of solar activity over hundreds and even thousands of years.

## Methods

For the simulation presented here, the full set of global, nonlinear MHD shallow-water equations^23^ have been employed, but in a rotating frame of reference and a pseudo-spectral algorithm^8,16^ has been implemented to solve them in a spherical polar coordinate for the solar tachocline. Due to the thinness of the solar tachocline (~0.04 R) compared to the solar radius (R) at tachocline depth, the divergence of radii and the density variation within the shallow fluid layer are ignored in the momentum and mass-continuity equations; therefore the shallow water approximation works well for the solar tachocline^7^. The horizontal velocity and magnetic field components are functions of latitude and longitude but independent of the vertical coordinate, whereas the vertical velocity and the vertical magnetic field are linear functions of height.

The parameters of the model are: (i) the “effective gravity” (G) of the tachocline, (ii) the latitudinal differential rotation amplitude (s), (iii) the peak field strength of toroidal magnetic bands and (iv) the latitude locations of the peak field. From helioseismic observations, the reference state differential rotation (*ω*) in the rotating frame is *ω* = ${s}_{0}-{s}_{2}{\mu }^{2}-{s}_{4}{\mu }^{4}-{\omega }_{c}$, in which *s*_0_, *s*_2_, *s*_4_ are the numerical coefficients to represent the solar latitudinal differential rotation, and *μ* is the sine latitude (see, e.g., helioseismically determined differential rotation^32^). Here *ω*_*c*_ represents the core rotation rate, which is approximately equal to the tachocline rotation rate at 32° latitude. Therefore, a value of *s*_0_ is chosen in such a way as to have *ω* = 0 at 32° latitude in the rotating frame of reference. Generally *S*_0_ is slightly more than 1, in our present case shown in Fig. 3, ${s}_{0}\simeq 1.05$. Thus the amplitude (s) of the pole-to-equator differential rotation is given by $({s}_{2}+{s}_{4})/{s}_{0}.$ The effective gravity, *G*_*eff*_, is a dimensionless parameter of the system. It can be related to the actual gravity as ${G}_{eff}=\frac{\delta \times \,{g}_{actual}\times \,H}{{({r}_{0}\times {\omega }_{c})}^{2}}$, where *g*_*actual*_ is the Sun’s dimensional gravity at the tachocline depth, *δ*, the fractional departure of the stratification from adiabatic, *H* the thickness of the unperturbed tachocline fluid shell, *r*_0_ the solar radius at tachocline depth, and *ω*_*c*_ the solid rotation rate of the solar core just beneath the tachocline. Note that *G*_*eff*_ is ${G}_{actual}\,\times \,\delta$, in which dimensionless tachocline gravity, ${G}_{actual}=\frac{{g}_{actual}\times \,H}{{({r}_{0}\times {\omega }_{c})}^{2}},$ and *δ* varies within 10^−5^ and 10^−1^. As discussed in an earlier paper^7^, the range of *δ* falls within 10^−5^–10^−4^ in the convective overshoot part of the tachocline (i.e. the top part of the tachocline above the base of the convection zone), and in the radiative part (below the base of the convection zone)the tachocline has a *δ* of ~10^−1^. Adiabatic stratification means *δ* = 0, hence zero effective gravity. In the tachocline depth, *G*_*eff*_ can vary from 0.01 to 100 in the entire tachocline^5^. The lower is the *G*_*eff*_ value, the more deformation of the free top surface is possible.

Here *r*_0_ and 1/*ω*_*c*_ are respectively the unit dimensionless length and time. Thus 100 dimensionless time units corresponds to about 1 year.

To prescribe the banded toroidal magnetic fields, which are the spot-producing fields, we use a Gaussian profile^5^ in latitude, as used by many authors previously. The Gaussian representation has parameters to prescribe the peak field strength of the magnetic band, latitude location of the band’s peak and the latitudinal width of the band, which describes how narrow or broad the spot-producing fields are at the tachocline depth. We consider 10-degree toroidal magnetic bands of opposite polarity in north and south hemispheres with peak field of 100 kGauss, placed at 30-degree latitudes of each hemisphere.

The computation of energies ($\bar{K}$, *K′*, $\bar{P}$, *P*′) are performed by following the volume integral formula^16^ given in expression (12). $\bar{K}$, $\bar{P}$ represent the axisymmetric parts (m = 0) of the energies, and *K*′, *P*′ the nonaxisymmetric (m > 0) parts.

### Data availability

All simulation data are stored in NCAR’s HPSS system. The model-outputs are obtained in the form of spectral coefficients, from which the detailed latitude-longitude planforms and longitude-averaged patterns can be derived. Simulation data and corresponding graphics routines will be available from NCAR’s High Altitude Observatory storage system for community use.
